# Does Group Size Matter for Behavior in Online Trust Dilemmas?

**DOI:** 10.1371/journal.pone.0166279

**Published:** 2016-11-29

**Authors:** Sabrina Artinger, Nir Vulkan

**Affiliations:** 1 Center for Adaptive Behavior and Cognition, Max Planck Institute for Human Development, Berlin, Germany; 2 Saïd Business School, University of Oxford, Oxford, United Kingdom; Universidad de Zaragoza, SPAIN

## Abstract

How does group size influence behavior in online trust dilemmas? We investigate cooperation in groups of 4 to 100 players. While overall levels of cooperation are stable across group sizes, we find significant gender differences: women increase cooperation with group size and cooperate significantly more than men in large groups. These results are robust when controlling for risk aversion, age, and other individual differences. They highlight the importance of studying behavior and gender differences in large groups.

## Introduction

The emergence of new technologies has dramatically reduced costs of coordinating cooperation of large groups. Yet, empirical research on cooperation has primarily focused on the interaction of small groups. Little is known about online cooperation and how group size impacts people’s willingness to trust in anonymous online interaction. 97% of the studies on cooperation involved groups of 2 to 10 people—with more than half of these studies considering dyadic interactions [1]. Only two studies investigated behavior in groups of 100 people or more [2, 3] and neither of them studied behavior in online settings.

To address this research gap, we study the influence of group size in an online trust dilemma. In the trust dilemma, both cooperation and defection constitute possible equilibria. If enough people cooperate, it is best to cooperate as well. However, if not enough people cooperate, it is best to defect. Thereby the cooperative equilibrium yields higher payoffs while the non-cooperative equilibrium is less risky as it provides a safe payoff independent of the choices of others. People face the dilemma that they can only reach superior outcomes if they mutually trust each other. For games with more than 2 players most equilibrium selection theories predict that the non-cooperative equilibrium will be selected *regardless* of the number of players because it has a bigger basin of attraction [4].

In this paper, we experimentally test this prediction by investigating the influence of group size on the general level of cooperation in an online trust dilemma as well as on individual propensities to cooperate. The next section describes the experimental design and procedure followed by the results in section 3. Finally, we discuss the results and implications.

## Experiment and Data

We conducted an online experiment using OXLab, the online platform of the Oxford experimental Laboratory for social science experiments (https://www.oii.ox.ac.uk/projects/oxlab/). OXLab provides a large data base of people with a broad range of backgrounds who registered to take part in online experiments. At OXLab ethical review is standardized for conventional socioeconomic experiments such as this one. This implies that the treatment of participants was in agreement with the ethical guidelines of the Central University Research Ethics Committee of the University of Oxford (http://www.admin.ox.ac.uk/curec/. The Chair of the Social Sciences Interdivisional Research Ethics Committee at that time was Professor Colin Mayer. The full list of Members can be found under https://www.admin.ox.ac.uk/councils/governance/committees/committeemembership/centraluniversityresearchethicscommitteemembership/). Specifically, all participants gave their informed consent to participate voluntarily, assuring them that analyses and publication of experimental data would be without an association to their real identities. As the experiment was conducted online, participants gave their consent online as part of the process of registering to the experimental database of OXLab. The experiment involved no deception of participants. As in other socioeconomic experiments, there were no additional ethical concerns.

The experiment was run in two sessions with a total of 135 participants (76 participants in the first session and 59 in the second session). For signing in on time, each participant received £5 which was added to his or her experimental account at the beginning of the experiment. Participants were then given general instructions and the experiment started. Participants played six rounds of the trust dilemma game without feedback. In each round they were asked to decide individually whether or not they want to cooperate and invest £5 into a group project. The project generated profits if more than 2/3 of the players in the group participated. In case fewer players participated, cooperators made a loss. Gains from cooperating were larger the higher the number of people cooperating. The payoffs of defectors were £0 independent of the choices of others; no free rider incentives existed. (The total payoff of defectors from participating in the study was £5 (= £5 they received for signing in on time plus £0 from the cooperation experiment). The payoffs of cooperators were given by:

$$f\left(x\right)=\{\begin{array}{ll} -5+\frac{15}{2}x & if\;0\leq x\leq \frac{2}{3}\\ 15x & if\;\frac{2}{3}\;<x\leq 1 \end{array}$$ (1)

where x is the number of cooperators around a given player.

In the instructions, payoffs were presented numerically and graphically to enable an easy understanding of the relation between the numbers of group members investing and the payoff from investing into the group project. Participants played six rounds of the trust dilemma without feedback. In each round, group size was randomly varied within subjects from 4 to 100 players (N = [4, 6, 8, 10, 20, 100]).

**Fig 1:**
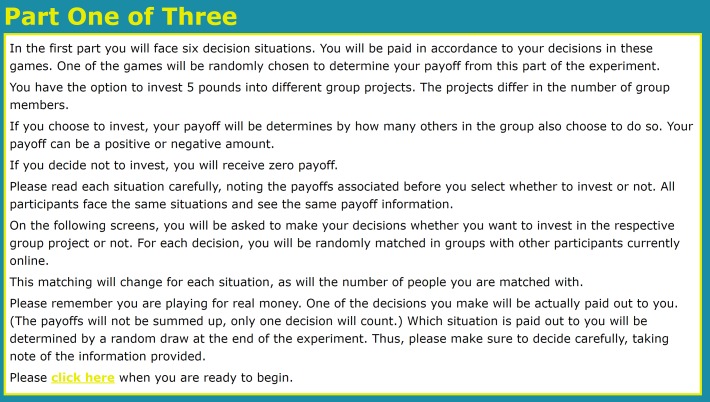
Screenshot—General instructions.

**Fig 2:**
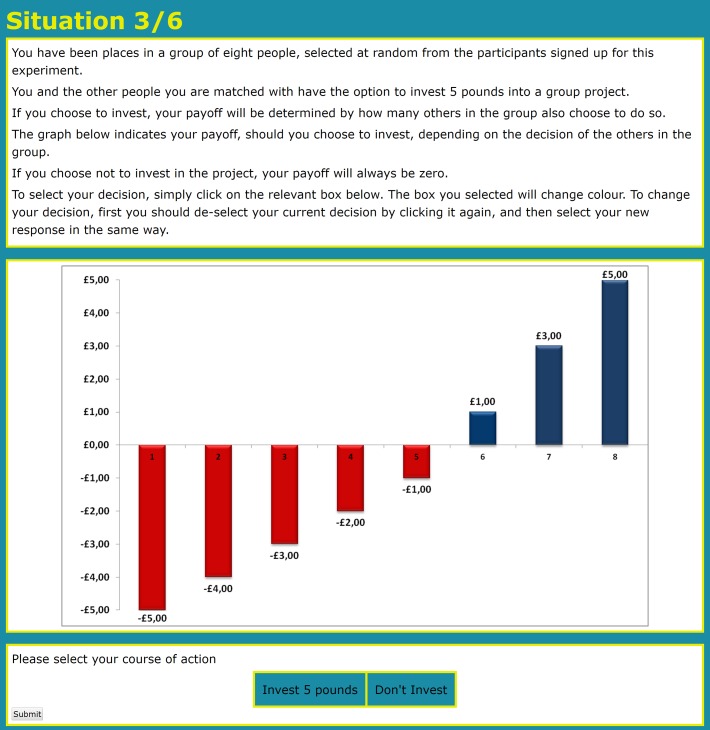
Screenshot—Instructions for N = 8.

Running online experiments requires a method that is robust against potential drop-outs and technical problems on the side of the participants. This is particularly relevant for experiments with large numbers of participants (Imagine an experiment with 100 participants and the internet connection of one of them is interrupted). An established method to assure that online experiments run smoothly is to process the actual matching of individuals only for determining the payoffs at the end of the experiment [5–7]. Since our experiment did not require feedback between rounds, we adopted this method and processed the actual matching only at the very end of the experiment to determine the payoffs by matching participants in groups of N players randomly selected from the pool of all 135 participants. The instructions are shown in Figs 1 and 2 below for the example of N = 8.

To test the robustness of our results, we controlled for gender, age, risk propensity, and other individual characteristics such as the Big Five personality characteristics and Locus of Control. The Five-Factor model of personality is a set of five broad trait dimensions often referred to as the “Big Five”: Extraversion, Agreeableness, Conscientiousness, Neuroticism, and Openness [8, 9]. Extraverted individuals are assertive and sociable, rather than quiet and reserved. Agreeable individuals are cooperative and polite, rather than antagonistic and rude. Conscientious individuals are task-focused and orderly, rather than distractible and disorganized. Neurotic individuals are prone to experiencing negative emotions, such as irritation, anxiety, and depression, rather than being emotionally resilient. Highly open individuals have a broad rather than narrow range of interests and prefer novelty to routine. The Big Five or five-factor model was developed to represent as much of the variability in individuals’ personalities as possible, using only a small set of trait dimensions. It has been widely accepted as an adequate taxonomy of personality traits and is frequently used to measure and control for personality traits [10].

The concept of Locus of Control refers to the extent to which individuals believe they can control events affecting them. A person's "locus" (Latin for "place" or "location") is conceptualized as either internal (the person believes they can control their life) or external (meaning they believe their decisions and life are controlled by environmental factors which they cannot influence, or by chance or fate) [11]. Locus of control is a widely-used concept personality research and generated much research in a variety of areas in psychology [12].

A standard limitation of online studies is that participation is almost always unsupervised. This is also the case for our study. Hence, we cannot be certain that participants indicated their true age and gender and that the decisions we observed were made by one individual alone.

After completing the six rounds of the trust dilemma, participants’ risk attitudes were measured via an incentivized lottery choice task [13] (see also S1 and S2 Screenshots. Holt and Laury Lottery Choice Task). Then they completed a questionnaire including gender, age and a personality inventory to measure the Big Five personality characteristics and Locus of Control. We used the 44-item version of the Big Five [14]. The extraversion subscale consisted of 8 items (α = 0.85; example item: “I see myself as someone who has an assertive personality”), the agreeableness subscale consisted of 9 items (α = 0.72; example item: “I see myself as someone who is helpful and unselfish with others”), conscientiousness was measured on a subscale of 9 items (α = 0.84; example item: “I see myself as someone who does a thorough job”), neuroticism on an 8 item subscale (α = 0.85; example item: “I see myself as someone who can be tense”), and the openness subscale had 10 items (α = 0.75; example item: “I see myself as someone who is curious about many different things”). For measuring Locus of Control we used the short version of the Rotter scale with six items (α = 0.63; example item: “I believe my success depends on ability rather than luck”). The internal consistency of this short version of the Rotter scale turned out to be below acceptable levels; i.e. Cronbach’s α < 0.70. For completeness, we included Locus of Control in the analyses despite the questionable internal consistency of the short scale. The results are, however, robust when leaving Locus of Control out. At the end of each session, an overview of the choices was given and participants indicated their preference for either receiving their payoff as a check or a voucher. At the end of the experiment, when both sessions were completed and the payoffs had been determined, participants received an email with their payoff either as a check or a voucher.

## Results

Contradicting predictions of equilibrium selection theories, a majority of people chose to cooperate. Average cooperation rates were relatively stable across different group sizes—ranging from 66% to 72% (see Table 1).

**Table 1:** Cooperation rate and average number of cooperators.

Group Size	4	6	8	10	20	100
Cooperation rate	0.68	0.67	0.72	0.66	0.70	0.69
AV # cooperators	2.72	4.04	5.75	6.59	14.08	68.90

**Fig 3:**
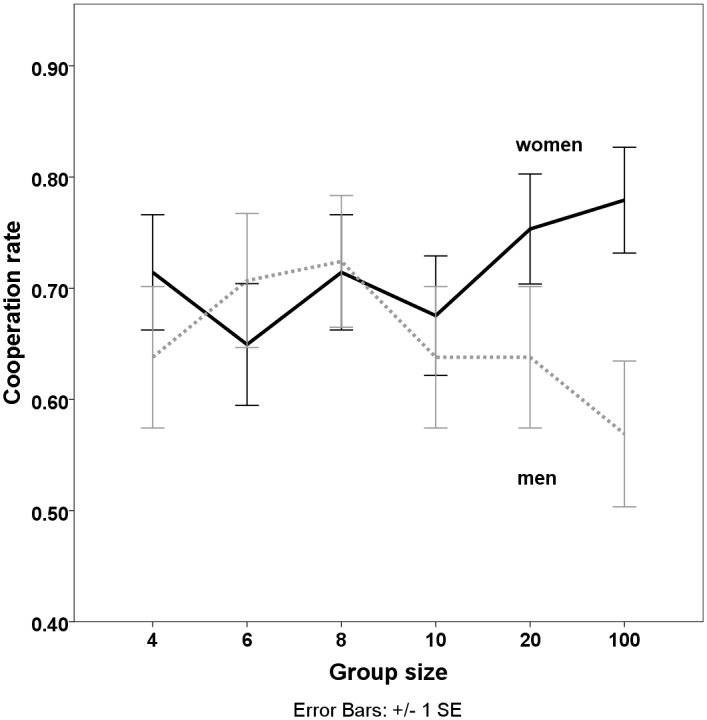
Cooperation rate of men and women as a function of group size. To control for *risk aversion*, *age*, and personality (*extraversion*, *agreeableness*, *conscientiousness*, *neuroticism*, *openness*, *and locus of control*) we ran several random effects logistic regressions. The descriptive statistics for the control variables are summarized in Table 2. Correlations of the independent variables can be found in Table 3. Results of the regression analyses are reported in Table 4.

Interestingly, we find that men and women differed significantly in their reactions to group size: women cooperated significantly more with increasing group size while men cooperated most in groups of 8 and least in groups of 100 players. Fig 3 below illustrates this interaction effect. For smaller group sizes, gender differences are less pronounced and statistically insignificant but they are significant for large groups of 20 or 100 group members. At a group size of 20, cooperation rates of men (M = 63.7%, SD = 0.485) and women (M = 75.3%, SD = 0.434) differ by 11.6% (t(133) = 1.45, p = 0.07); at a group size of 100, this difference increases to 21% (men M = 56.9%, SD = 0.048; women M = 77.9%, SD = 0.066; t(133) = 2.66, p = 0.004).

**Table 2:** Descriptive statistics.

	Mean	Min	Max	Mean men	Mean women
*(1) Risk aversion*	5.72	0	10	5.88	5.61
*(2) Average Age*	30.73	21	55	30.35	31.03
*(3) Locus of Control*	2.61	-6	6	3.21	2.16
*(4) Extraversion*	25.19	10	39	24.97	25.49
*(5) Agreeableness*	33.55	21	45	34.12	33.12
*(6) Conscientiousness*	33.41	15	44	32.85	33.83
*(7) Neuroticism*	22.02	8	37	19.45	23.96
*(8) Openness*	37.46	18	50	36.95	37.84

Number of participants: 135 (Male: 58; Female: 77)

**Table 3:** Correlation matrix.

	(1)	(2)	(3)	(4)	(5)	(6)	(7)	(8)	(9)	(10)
*(1) Group size*	1.0000									
*(2) Gender*	-0.0000	1.0000								
*(3) Risk aversion*	0.0018	0.0443	1.0000							
*(4) Age*	0.0000	-0.0354	**-0.0925**	1.0000						
*(5) Locus of Control*	0.0000	**0.1699**	**0.2074**	**0.1075**	1.0000					
*(6) Extraversion*	-0.0000	-0.0559	-0.0527	-0.0447	**0.3252**	1.0000				
*(7) Agreeableness*	-0.0000	**0.0967**	**-0.0927**	0.0389	**0.2256**	**0.2342**	1.0000			
*(8) Conscientiousness*	-0.0000	**-0.0845**	-0.0203	**0.1137**	**0.3912**	**0.3065**	0.3249	1.0000		
*(9) Neuroticism*	0.0000	**-0.3603**	0.0294	**-0.1176**	**-0.4766**	**-0.2325**	**-0.3772**	**-0.4095**	1.0000	
*(10) Openness*	0.0000	-0.0781	-0.0566	-0.0811	0.0496	0.0783	0.0099	0.0684	0.0353	1.0000

**Table 4:** Random effects logistic regressions.

Model	(1)	(2)	(3)	(4)
*Groupsize*	0.000 (0.003)	0.007* (0.004)	0.009* (0.005)	0.009* (0.005)
*Gender*	-0.459 (0.374)	-0.106 (0.402)	-0.192 (0.511)	-0.227 (0.547)
Gender*groupsize		-0.015***(0.006)	-0.021***(0.007)	-0.021***(0.007)
*Risk aversion*			0.079 (0.673)	0.112 (0.105)
*Age*				0.400* (0.194)
*Locus of Control*				-0.021 (0.098)
*Extraversion*				0.048 (0.042)
*Agreeableness*				-0.063 (0.049)
*Conscientiousness*				0.059 (0.048)
*Neuroticism*				-0.056 (0.049)
Openness				0.024 (0.040)
Constant	1.406***(0.267)	1.256***(0.275)	1.077 (0.673)	-0.749 (3.365)
ln σ_u_^2^	1.151 (0.241)	1.182 (0.240)	1.534 (0.261)	1.367 (0.268)
σ_u_	1.778 (0.214)	1.806 (0.217)	2.154 (0.281)	1.981 (0.265)
Rho	0.490 (0.060)	0.498 (0.060)	0.585 (0.063)	0.544(0.066)
χ^2^	1.52	8.16	12.49	23.70
AIC	887.24	882.32	693.96	694.77
Number of observations	810	810	678	678
Number of participants	135	135	113	113

*Notes*: 22 of the 135 participants exhibited inconsistent response patterns in the Holt & Laury (2002) task [13]. We therefore excluded these participants from model (3) and (4) which control for risk aversion. Although not reported here, we also ran further models without controlling for locus of control which has not reached satisfying levels of internal consistency in our sample. The results remain unchanged.

*** Significant at the 1 percent level.

** Significant at the 5 percent level.

* Significant at the 10 percent level.

The results show that *groupsize* had a positive main effect on cooperation only when controlling for the interaction effect of gender*groupsize. *Gender* had no significant main effect, but the interaction effect of gender*groupsize is highly significant; men cooperated less in bigger groups, women cooperated more in bigger groups. This finding is robust also when controlling for risk aversion, age, and personality. Interestingly, risk aversion as measured in accordance to Holt and Laury (2002) did not affect behavior. Age on the other hand had a substantial positive influence on cooperation—older people cooperated more. Furthermore, the Big Five personality factors did not significantly affect behavior.

Analyzing individual strategies in reaction to group size, we identified five different groups:

- Group 1 (always cooperate): participants who always cooperated
- Group 2 (always defect): participants who always defected
- Group 3 (switching from cooperate to defect): participants who cooperated in smaller groups but defected in larger groups
- Group 4 (switching from defect to cooperate): participants who defected in smaller groups but cooperated in larger groups
- Group 5 (other): participants who did not exhibit a systematic behavioral pattern depending on group size

Table 5 shows the distribution of men and women in each group. More women than men always cooperated while more men than women always defected. Consistent with the interaction effects reported above, there were more men than women who switched from cooperating to defecting when the group size increased, and more women than men who switched from defecting to cooperating when group size increased.

**Table 5:** Overview strategies.

Group	Strategy	Total	Women	Men
1	Always cooperate	36.3% (49/135)	61.2% (30)	38.8% (19)
2	Always defect	7.4% (10/135)	30.0% (3)	70.0% (7)
3	Switching from cooperate to defect	9.6% (13/135)	38.5% (5)	61.5% (8)
4	Switching from defect to cooperate	12.6% (17/135)	64.7% (11)	35.3% (6)
5	Other strategies	34.1% (46/135)	56.5% (26)	43.5% (20)

There were more women than men whose behavior is consistent with generally trusting into the cooperativeness of their group members and with trusting into the cooperativeness of larger groups but not of smaller groups. On the contrary, there were more men than women whose behavior is consistent with a generally distrusting in the cooperativeness of their group members, and with trusting more into the cooperativeness of smaller groups while not trusting larger groups.

## Discussion and Conclusion

While our results show no significant impact of group size on mean cooperation rates we find significant gender differences in reactions to group size. These differences were largest in groups of 100, resulting in differences in cooperation rates of 21%. Results show that these gender differences are robust and cannot be explained by differences in risk attitudes or general personality traits.

Our individual level analysis and results from studies on gender differences in economic and social behavior indicate that the observed differences might be rooted in participants’ construal of the situation; particularly in the construal of changes in group size.

An integrative framework suitable to study whether men and women differ systematically in their construal of trust dilemma type of situations is interdependency theory [15–17]. Rooted in game theory, interdependency theory assumes that interaction is a combined function of the objective incentive structure (e.g., the structure of the respective game), the interacting parties which transform the objective structure and construct an effective structure of subjective outcomes, and the interaction dynamics. We believe that in our trust dilemma, the construal of subjective outcome structures involves two factors that are plausible drivers of the observed sex difference in behavior; first, the focus on the absolute number of cooperators versus focus on the absolute number of defectors and second, the perceived responsibility for the other group members. First, constructing subjective outcome structures, individuals might either focus on the absolute number of cooperators necessary for effective cooperation or on the absolute number of defectors that can be tolerated without destroying the effectiveness of cooperation. Focusing primarily on the absolute number of cooperators necessary for effective cooperation is consistent with switching from cooperating to defecting when group size increases. The more people a person needs to trust in, the higher the potential threat of defection might appear. Focusing instead on the absolute number of defectors that can be tolerated without destroying effective cooperation is consistent with switching from defecting to cooperating when group size increases. For people focusing on “defection tolerance”, larger groups might appear less threatening as the absolute number of defectors can be relatively large (in our game 33 people out of 100) without making cooperation unproductive. Following these perspectives, in our study women’s behavior would be consistent with a focus on defection tolerance while men’s behavior would be consistent with a focus on the number of people to trust in. Such a perspective could be related to results showing that women are more protective and focused on avoiding losses than men (see for example [18] for a survey in Psychology, and [19, 20] for surveys in Economics).

Second, in large groups the harm of defecting affects a larger number of other people than in small groups. Thus, people with other regarding preferences might perceive a stronger incentive to cooperate in larger groups. This incentive from other regarding preferences might be even strong enough to overcome the lack of trust in the cooperativeness of others. Constructing the presented situation as a tradeoff between other-regarding and monetary incentives, larger groups would imply higher levels of cooperation for those with other-regarding preferences. Our data would be consistent with more women than men constructing the presented situation in this way.

So given that women cooperated more than men in large groups, should we encourage more women to engage in large group interaction to achieve better social outcomes? The success of micro credit institutions building on mutual trust within large women networks such as the Grameen Bank (http://www.grameen-info.org), which gives loans exclusively to women, might be indicative of this. Other domains where these effects could have an important impact are collective political action, crowd funding and community work.

As Arrow (1974, p. 26) notes “the agreement to trust each other cannot be bought” [21]. It is thus inevitable to understand the factors and processes that shape trust behavior and to identify situations in which people—men and women—are more inclined to trust in the cooperativeness of anonymous others. This paper highlights the importance of understanding the impact of group size on cooperation and aims to encourage future research on this topic.

## Supporting Information

S1 Dataset (CSV)

S1 Screenshot Holt and Laury Lottery Choice Task part I. (TIF)

S2 Screenshot Holt and Laury Lottery Choice Task part II. (TIF)

## References

[1] Balliet D, Li N, Macfarlan S, Van Vugt M. Sex Differences in Cooperation: A Meta-Analytic Review of Social Dilemmas, Psychol Bull. 2011; 137(6): 881–909. doi: 10.1037/a0025354 21910518 10.1037/a0025354

[2] Wit A, Wilke H. Public good provision under environmental and social uncertainty. Eur J Soc Psychol. 1998; 28: 249–256.

[3] Poppe M. The specificity of social dilemma situations. J Econ Psychol. 2005; 26: 431–441.

[4] Kim Y. Equilibrium Selection in n-Person Coordination Games. Games Econ Behav. 1996; 15(2): 203–227.

[5] Horton J.J., Rand D.G., Zeckhauser R.J. The online laboratory: conducting experiments in a real labor market. Exp Econ. 2011; 14(3): 399–425.

[6] Cooper D.J., Saral K.J. Entrepreneurship and team participation: an experimental study. European Economic Review. 2013; 59:126–140.

[7] Hergueux J., Jacquemet N. Social preferences in the online laboratory: a randomized experiment. Exp Econ. 2015; 18(2): 251–283.

[8] Digman JM. Personality structure: Emergence of the five-factor model. Annual Review of Psychology. 1990; 41, 417–440.

[9] McCrae RR., John OP. An Introduction to the Five-Factor Model and its Applications. Journal of Personality. 1992: 60(2), 175–215. 10.1111/j.1467-6494.1992.tb00970.x 1635039

[10] McCrae RR, Costa PT. Personality in adulthood: a five-factor theory perspective- 2nd edition 2003 New York: The Guildford Press.

[11] Rotter JB. Generalized expectancies for internal versus external control of reinforcement. Psychological Monographs: General & Applied. 1966: 80(1), 1–28. 5340840

[12] Lefcourt HM. Locus of Control: Current Trends in Theory & Research- 2nd edition 2014 New York: Psychology Press.

[13] Holt CA, Laury S. Risk Aversion and Incentive Effects. Am Econ Rev. 2002; 92(5): 1644–1655.

[14] John OP, Donahue EM, Kentle RL. The “Big Five” Inventory-Versions 4a and 54. Technical Report. IPAR. University of California Berkeley; 1991.

[15] Kelley HH, Thibaut JW. Interpersonal relations: A theory of interdependence. New York: Wiley; 1978.

[16] Kelley HH, Holmes JW, Kerr NL, Reis HT, Rusbult CE, Van Lange PAM. An atlas of interpersonal situations. New York: Cambridge; 2003.

[17] Van Lange PAM, Rusbult CE. Interdependence theory In Van Lange PAM, Kruglanski AW and Higgins ET (Eds.), *Handb Theories Soc Psychol*, 251–272. Thousand Oaks, CA: Sage; 2012.

[18] Byrnes JP, Miller DC, Schafer WD. Gender differences in risk taking: A meta-analysis. Psychol Bull. 1999; 125(3): 367.

[19] Eckel CC, Grossman PJ. Men, women and risk aversion: Experimental evidence. Handb Exp Econ Res. 2008; 1: 1061–1073.

[20] Croson R, Gneezy U. Gender differences in preferences. J Econ Res Lit. 2009; 47(2): 448–474.

[21] Arrow K. The limits of organization. W.W. Norton & Comp. Inc New York; 1974.

