# Supplementary figures and images for: Does Group Size Matter for Behavior in Online Trust Dilemmas?
Source: PLoS One. 2016 Nov 29;11(11):e0166279. doi: 10.1371/journal.pone.0166279 (PMC5127509; doi:10.1371/journal.pone.0166279)

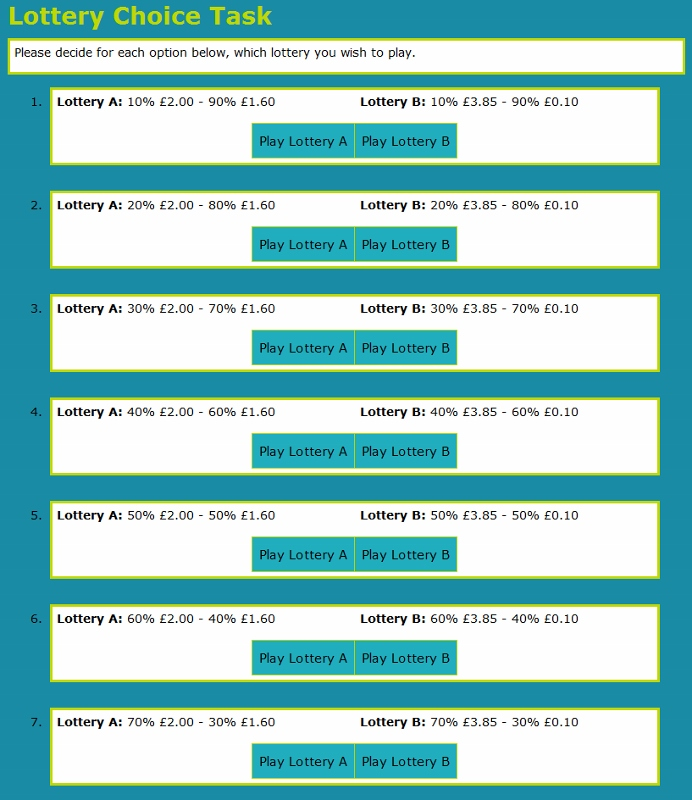

Supplement: S1 Screenshot — (TIF) [file pone.0166279.s002.tif]

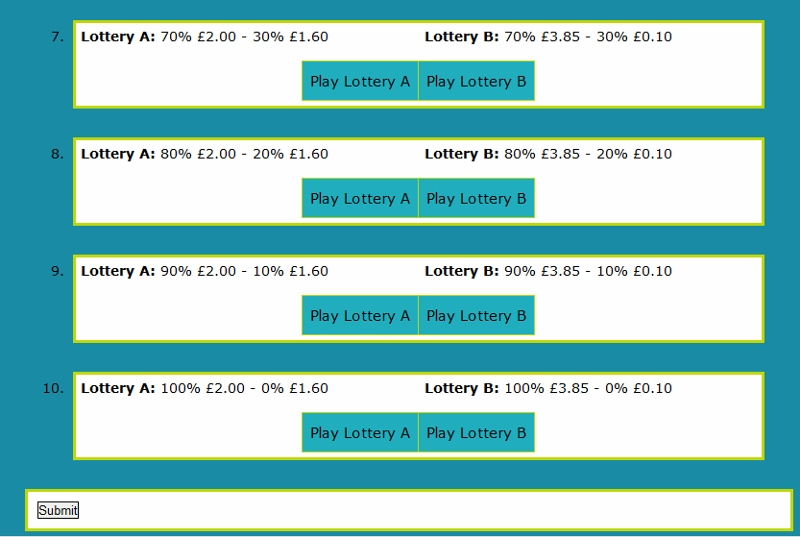

Supplement: S2 Screenshot — (TIF) [file pone.0166279.s003.tif]
